# Are gamifying exergames the future of intervention for people with dementia? Systematic review and meta-analysis

**DOI:** 10.3389/fnhum.2026.1870647

**Published:** 2026-07-27

**Authors:** Elena Papamichael, Irene-Chrysovalanto Themistocleous, Stelios Hadjisavvas, Christina Michailidou

**Affiliations:** Department of Health Sciences, School of Life and Health Sciences, University of Nicosia, Nicosia, Cyprus

**Keywords:** activities of daily living, cognitive function, dementia, exergaming, motor function

## Abstract

**Background:**

Gamified exergaming has emerged as a promising approach for enhancing cognitive and motor function in older adults, yet its effectiveness in people with dementia remains unclear. Existing research has largely focused on individuals with mild cognitive impairment, leaving a gap in understanding how these interventions perform in populations with more advanced cognitive decline.

**Objective:**

The primary goal of this study was to systematically evaluate and quantify the effects of gamified exergaming on cognitive function. Secondary, to evaluate motor performance, and activities of daily living (ADL) in individuals with dementia compared with non-exergaming or standard care interventions.

**Methods:**

A systematic search of MEDLINE/PubMed, Cochrane CENTRAL, and EBSCO (2016–2026) identified randomized controlled trials involving exergaming interventions for people with dementia. Nine studies met the inclusion criteria. Risk of bias was assessed using the RoB2 tool. Meta-analyses were conducted using random-effects models, with outcomes synthesized for MoCA, MMSE, ADL, and motor function (SPPB).

**Results:**

A total of 717 participants were included. Exergaming produced a significant improvement in global cognition measured by the MoCA (*p* = 0.007). No significant effects were observed for MMSE (*p* = 0.065), ADL performance (*p* = 0.489), or motor function assessed with the SPPB (*p* = 0.182). Considerable heterogeneity was present in motor outcomes. Most interventions used non-immersive VR platforms, with substantial variation in duration, frequency, and training intensity.

**Conclusion:**

Gamified exergaming demonstrates a meaningful positive effect on global cognition in individuals with dementia, while evidence for motor and functional improvements remains inconclusive. The variability of intervention protocols and limited number of dementia-specific trials highlight the need for standardized, high-quality randomized studies. Exergaming represents a feasible and engaging therapeutic option that may complement traditional rehabilitation in dementia care.

## Introduction

Dementia is a progressive neurodegenerative condition that is characterized by motor and cognitive impairments, limiting functionality, independence and quality of life (Gale et al., 2018). Global estimates indicate that over 57 million individuals were living with dementia in 2025, reflecting the substantial and growing public health burden associated with aging populations (Nandi et al., 2024). Evidence from recent epidemiological reviews highlights that dementia incidence varies significantly across regions, with declines observed in parts of Europe and the United States but increases reported in countries such as Japan, Taiwan, and Republic of Korea, underscoring the heterogeneity of global trends (Vella et al., 2026). Low- and middle-income countries are expected to experience the most rapid growth in dementia cases over the coming decades, largely due to demographic expansion and under-diagnosis in earlier years (Nandi et al., 2024).

Dementia is primarily characterized by progressive cognitive decline that interferes with independence in everyday activities. Core cognitive impairments typically include memory deterioration, especially episodic memory, executive dysfunction, language disturbances, and visuospatial deficits (Dowllah et al., 2023). memory impairment remains the most prevalent early symptom, reflecting degeneration in medial temporal lobe structures, while deficits in planning, attention, and problem-solving emerge as frontal networks become affected (Qin et al., 2025). Furthermore, visuospatial dysfunction such as difficulty navigating familiar environments or recognizing objects, have been identified as a common early sign, particularly in Alzheimer’s disease and dementia with Lewy bodies (Mukadam et al., 2024).

Beyond cognitive decline, motor symptoms are increasingly recognized as integral to the clinical presentation of several dementia syndromes. Bradykinesia, impaired gait and dual-task performance, and balance disturbances often appear as the disease progresses, reflecting broader neurodegenerative involvement leading to functional impairment (Liampas et al., 2025; Andrade-Guerrero et al., 2024). Understanding these cognitive and motor impairments is essential for identifying the functional challenges faced by individuals with dementia. These challenges directly inform the selection and design of therapeutic interventions aimed at preserving independence and improving overall quality of life.

Traditional rehabilitation approaches presented improvement in various outcomes. On the other hand, traditional interventions often struggle to increase the motivation of the people or to maintain engagement during the therapies (Saúde et al., 2023; Forstmeier et al., 2025). Virtual reality (VR) is a promising tool for enhancing cognitive and motor training. Interventions based on different types of VR, enriched, interactive tasks that can simulate real-world activities, provide real time feedback, and promote neuroplasticity through repetitive, goal oriented practice (Lukacs et al., 2022). VR provides people with the ability to implement various activities, both motor and cognitive, leading to an increase in motivation and engagement, where multiple repetitive tasks can be completed.

Interest in virtual reality–based rehabilitation has grown rapidly, with most existing evidence concentrating on individuals with mild cognitive impairment (Yang et al., 2025; Yuan et al., 2025). This highlights the need to examine how advanced technologies perform in populations with moderate to severe cognitive decline, such as people diagnosed with dementia, who may demonstrate different therapeutic responses and levels of usability. Moreover, the literature presents substantial variation in intervention characteristics such as dosage, frequency, and delivery methods, as well as methodological approaches (Liao et al., 2019; Robert et al., 2014), reinforcing the importance of systematic evaluation and critical review.

Furthermore, virtual-reality based rehabilitation has gained considerable attention in recent years, the majority of published evidence continue to focus on individuals with mild cognitive impairment (MCI) rather than those with a formal diagnosis of dementia (Lin et al., 2022; Morán et al., 2024). Recent systematic reviews show that VR interventions are predominantly tested in MCI populations, where cognitive demands, usability, and therapeutic responsiveness differ substantially from those in people with moderate or severe cognitive decline (Maggio et al., 2025a; Abd-Alrazaq et al., 2022). As a result, the applicability of these findings to dementia remains limited, leaving a critical gap in understanding how advanced technologies perform in populations with more pronounced neurodegeneration. Furthermore, several reviews emphasize that dementia specific VR studies are scarce, often underpowered, and methodologically heterogeneous, making it difficult to draw firm conclusions about their clinical effectiveness (Kim et al., 2019). This gap underscores the necessity of conducting targeted research that evaluates VR-based interventions directly in dementia populations, where therapeutic needs, cognitive load, and motor impairments differ markedly from those with milder conditions.

Accordingly, conducting this systematic review and meta-analysis is crucial for synthesizing the existing evidence and addressing the current gaps regarding the effects of VR-based interventions on cognitive, motor, and functional outcomes in individuals with dementia. By providing rigorous, evidence-based conclusions, this review will support healthcare professionals in selecting appropriate VR techniques and outcome measures, while also offering the wider community accessible insights into rehabilitation approaches that may enhance the quality of life for people living with dementia.

### Purpose of study

This systematic review and meta-analysis aims to identify, extract, and synthesize data from the included studies. Its primary objective was to evaluate the effects of exergaming-based rehabilitation on cognitive function in individuals with dementia compared with non-exergaming interventions or standard care. In addition, the review assesses the influence of exergames on motor performance and activities of daily living (ADL) within this population.

## Methods

### Search strategy

**Figure 1:**
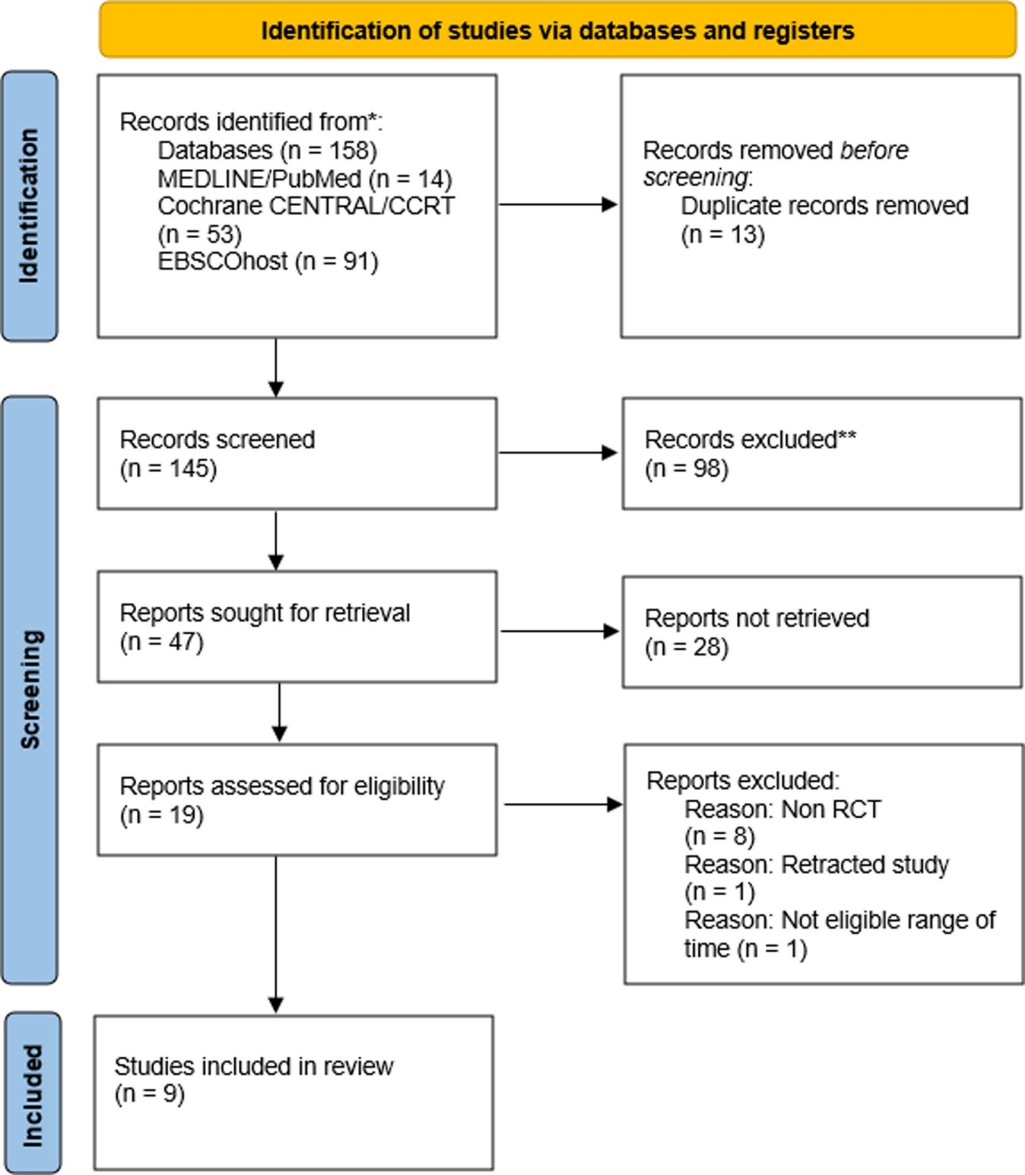
Search strategy flow diagram.

This systematic review was prospectively registered in the PROSPERO database (CRD420251253281). Its conduct adhered to the 2020 PRISMA guidelines, applying the Population, Intervention, Comparison, and Outcomes (PICO) framework. A comprehensive search was carried out across the MEDLINE/PubMed, Cochrane CENTRAL/CCRT, and EBSCOhost Research Databases (Academic search Ultimate, APA, CINAHL Plus, Health Source, Psychology and Behavioral Sciences Collection) with restrictions on publication date (2016–2026) and language (English). Only English-language articles were included due to resource constraints for translation and to ensure accurate interpretation of methodological details and outcome measures. The time frame 2016–2026 was chosen to capture contemporary exergaming and VR-based interventions, reflecting rapid technological developments and current clinical practice. A representative search string was: ((((((dementia[Title/Abstract]) OR (major cognitive impairment[Title/Abstract])) OR (Alzheimer[Title/Abstract])) AND ((((((exergaming[Title/Abstract]) OR (exergames[Title/Abstract])) OR (VR[Title/Abstract])) OR (virtual reality[Title/Abstract])) AND ((((rehabilitation[Title/Abstract]) OR (exercises[Title/Abstract])) AND ((((((cognitive function[Title/Abstract]) OR (cognition[Title/Abstract])) OR (motor function[Title/Abstract])) OR (quality of life[Title/Abstract])) OR (QOL[Title/Abstract])) Filters: Randomized Controlled Trial. The full search process is illustrated in Figure 1.

### Inclusion criteria

The inclusion criteria were as follows: (1) randomized controlled trials (RCTs); (2) diagnosis of Dementia; (3) the experimental intervention to have used ER that included exergaming tools. [This should include non-immersive tools, semi-immersive tools, and fully immersive tools]; (4) the control group to have practiced in any other physiotherapy program or standard care; (5) Studies were required to report cognitive outcomes as a primary inclusion criterion. Trials that additionally assessed motor function or ADL were also eligible. When motor or ADL measures were available, they were extracted and synthesized as secondary outcomes. Published studies from 2016–2026 were included.

### Interventions

#### Exergaming

Exergaming is defined as any technology-mediated activity that combines physical exercise with interactive, game-based elements. Such interventions require active bodily movement to advance through game tasks and provide real-time feedback, scoring, or challenge progression (Ouanes, 2023).

#### Non-immersive VR

Non-immersive VR involves screen- or projector-based virtual environments where participants interact using body movements, handheld controllers, or pressure platforms while remaining fully aware of their physical surroundings (Shah et al., 2022).

#### Semi-immersive VR

Semi-immersive VR uses large projection systems, multi-screen setups, or cave-like environments that partially surround the user, and increase the sense of presence. The participant remains physically grounded in the real world but experiences a more enveloping visual field than in non-immersive systems (Martin-Niedecken and Schättin, 2020).

#### Fully immersive VR

Fully immersive VR requires head-mounted displays (HMDs) that block out the physical environment and create a 360 ° virtual space. These systems provide the highest level of presence and sensory immersion, often incorporating motion tracking or handheld controllers (Martin-Niedecken and Schättin, 2020).

#### Non gamifying exercise rehabilitation

This approach encompasses traditional physiotherapy consisting of structured therapeutic exercise programs that lack gamified or interactive digital components. Interventions, such as strength, balance, aerobic, or functional training, are guided by a therapist or performed using standard exercise equipment, eschewing the game mechanics, scoring systems, or virtual environments characteristic of exergaming (Taylor et al., 2007).

### Quality assessment of studies

Study selection was conducted by two independent reviewers, but at different time points. During the initial search in January 2026, IC-T screened titles and abstracts, and EP independently screened the full texts of all potentially eligible studies. Following the updated search in April 2026, the same two reviewers again independently screened the newly identified records. If any disagreement arose, SH provided the final decision based on the eligibility of the articles, after screening. No discrepancies were found during the study quality assessment. After final study inclusion, EP extracted all study characteristics and outcome data. SH independently checked the extracted data for accuracy and completeness.

### Risk-of-bias assessment

**Figure 2:**
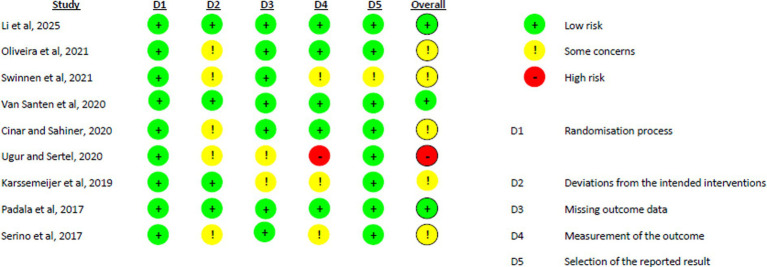
Assessment of risk of bias through RoB2.

For the evaluation of risk of bias, two independent reviewers used the Cochrane risk of bias tool (RoB2). EP and CM assessed the risk of bias, and results are presented in Figure 2.

### Data synthesis and analysis

Meta-analytic procedures were conducted using OpenMeta-Analyst, which applied a random-effects model to account for expected clinical and methodological variability across studies. For continuous outcomes, mean differences (MD) were calculated when studies used the same measurement scale, and standardized mean differences (SMD) when different scales were employed. Each study’s weight in the pooled estimate was determined by its inverse variance and the estimated between-study variance (*τ*^2^), calculated using the DerSimonian–Laird estimator.

Heterogeneity was assessed using the *I*^2^ statistic, which quantifies the proportion of variability attributable to between-study differences. The interpretation of *I*^2^ values was conducted according to conventional thresholds, with values above 75% indicating substantial heterogeneity. The *τ*^2^ estimate was also reported to reflect the absolute magnitude of between-study variance.

For the purposes of quantitative synthesis, all exergaming and VR-based interventions were pooled into a single experimental category. This approach was selected to evaluate exergaming as a unified therapeutic concept rather than differentiating between specific technological formats. Although non-immersive exergaming, semi-immersive VR, and fully immersive VR differ in immersion level and potential therapeutic mechanisms, the available evidence base was insufficient to support meaningful subgroup analyses. These distinctions and their implications are addressed in the Discussion.

Forest plots were generated to present pooled effect sizes with 95% confidence intervals, and statistical significance was set at *p* < 0.05.

## Results

### Study selection

The database search initially identified 158 records. After screening titles and abstracts, 19 studies were retained for full-text assessment. Ten of these were subsequently excluded: Eight did not meet this review’s eligibility criteria, one was not within the acceptable timeframe, and one study was retracted from the database. Following these exclusions, a total of nine studies fulfilled all inclusion criteria for the systematic review.

The Levene’s test used to assess the comparability of clinical characteristics across studies indicated that variance was homogeneous (*p* > 0.05), supporting the decision to proceed with the meta-analysis. Four of the group comparisons also demonstrated acceptable heterogeneity levels (*p* > 0.05), allowing pooled analyses for quality of life, activities of daily living, and physical function. In contrast, mental function (depression) did not meet the predefined heterogeneity threshold (*p* = 0.002) and was therefore excluded from further quantitative synthesis. The outcomes that satisfied the heterogeneity criteria and were meta-analyzed are summarized in Table 1.

**Table 1:** Results of the meta-analysis.

Study	Weights	MoCA
*Cognitive function*		
Li et al., 2025	40.95%	3.83 (CI: 1.04–6.61)
Swinnen et al., 2021	24.73%	(1.42)
Çinar and Sahiner, 2020	43.30%	*p* = 0.007

Study	Weights	MMSE
*Cognitive function*		
Oliveira et al., 2021	2.01%	1.30 (CI: −0.08–2.69)
van Santen et al., 2020	15.14%	(0.70)
Uǧur and Sertel, 2020	19.28%	*p* = 0.065
Serino et al., 2017	18.11%	
Padala et al., 2017	45.43%	

Study	Weights	Katz index
*ADL*		
Swinnen et al., 2021	14.05%	−0.29 (CI: −1.13–0.54)
Karssemeijer et al., 2019a	16.51%	(0.42)
Padala et al., 2017	69.33%	*p* = 0.48

Study	Weights	SPPB
*Motor function*		
Swinnen et al., 2021	31.65%	1.62 (CI: −0.76–4.00)
van Santen et al., 2020	34.38%	(1.21)
Karssemeijer et al., 2019a	33.96	*p* = 0.18

Significant statistical difference <0.05. CI, confidence interval.

### Bias assessment

The overall risk of bias assessment using the RoB2 tool indicated that the methodological quality of the included studies was generally robust, with most domains rated as low risk across the majority of trials. The randomization process (D1) and the measurement of outcomes (D5) were consistently judged as low risk, reflecting adequate allocation procedures and reliable reported results. A small number of studies demonstrated some concerns in domains such as deviations from intended interventions (D2) and reliability of measurement of outcomes (D4), suggesting minor uncertainties related to protocol adherence or selective outcomes (Swinnen et al., 2021; Serino et al., 2017; Karssemeijer et al., 2019a). Only isolated instances of high risk were observed, primarily linked to Measurement of the outcome (D4) (Uǧur and Sertel, 2020). Taken together, the evidence base shows a predominantly low to some risk of bias (Figure 2).

### Population characteristics

In this systematic review and meta-analysis, 717 participants with dementia were extracted from where the majority of them (52.8%) were females. A total of 342 participants were male (47.2%). The allocation of the participants was almost equal (49.94%) in the experimental group, and 50.06% in the control group. The mean age of the participants was 78.07(±6.03) (Table 2).

**Table 2:** Demographic characteristics.

Study	Country	Gender	Age	Disease stage
Li et al., 2025	China	Male = 99	72.7 ± 4.58	Moderate
		Female = 127		
Oliveira et al., 2021	Portugal	Male = 5	83 ± 5.66	Moderate
		Female = 12		
Swinnen et al., 2021	Switzerland	Male = 10	85 ± 6.05	Early/Moderate
		Female = 35		
Çinar and Sahiner, 2020	Türkiye	Male = 53	69.98 ± 7.67	Early
		Female = 67		
Uǧur and Sertel, 2020	Türkiye	Male = 27	73.44 ± 4.35	Early/Moderate
		Female = 9		
van Santen et al., 2020	Netherlands	Male = 60	79 ± 6.5	N/A
		Female = 62		
Karssemeijer et al., 2019a	Netherlands	Male = 62	79.9 ± 6.5	Early
		Female = 53		
Padala et al., 2017	United States	Male = 23	73 ± 6.2	Early/Moderate
		Female = 7		
Serino et al., 2017	Italy	Male = 3	86.62 ± 4.86	Early
		Female = 17		

### Intervention characteristics

All the included studies applied gamifying exergaming programs as a rehabilitation method for the experimental group. Eight studies used non-immersive VR programs (Li et al., 2025; Swinnen et al., 2021; Çinar and Sahiner, 2020; Oliveira et al., 2021; van Santen et al., 2020; Uǧur and Sertel, 2020; Padala et al., 2017; Karssemeijer et al., 2019a). Only one study used immersive VR tool for the rehabilitation (Serino et al., 2017). In this study, the frequency application of the program was 3 times per week, for 30 min per session. The duration of interventions ranged between 15 and 60 min. The total duration of the program was 12 weeks, with a range of 4–24 weeks (Table 3).

**Table 3:** Characteristics of the interventions.

Study	Participants (*N*)	Duration of intervention (minutes)	Frequency of intervention	Duration (weeks)	ER intervention	Non-ER intervention	Follow up
	Experimental/Non-experimental						
Li et al., 2025	226	60	2	12	Nintendo Switch game WarioWare (flexibility, joint mobility, coordination, and balance.)	Physical and mental exercises (board games, table-based interactions)	6 months
	113/113						
Oliveira et al., 2021	17	40	2	6	Computerized cognitive stimulation program with non-immersive VR	Treatment as usual	N/A
	10/7						
Swinnen et al., 2021	45	15	3	8	Non Immersive VR- Dividat Senso (steps, gait)	Music therapy, physical exercise (gait)	4 months
	23/22						
Çinar and Sahiner, 2020	120	15	2	12	Non Immersive VR (3 different 5 min computer games, and 3 min physical exercise video	Rivastigmine patch therapy	N/A
	60/60						
Uǧur and Sertel, 2020	32	30	2	6	Nintendo Wii virtual reality (soccer heading, tilt table, tightrope tension, perfect 10, cycling, tilt city, jogging plus, Hula-Hoop, step basics, and penguin slide.)	No treatment	N/A
	16/16						
van Santen et al., 2020	112	60	5	25	Non-Immersive VR Interactive cycling	Arts, craft, physical exercise	3 months
	73/39						
Karssemeijer et al., 2019a	115	50	3	12	Non-immersive VR (cognitive-aerobic bicycle training on a stationary bike connected to a video screen)	Aerobic training (cycling, relaxation and flexibility exercises)	6 months
	38/77						
Padala et al., 2017	30	30	5	16	Non-Immersive VR (Wii-Fit program: yoga, strength training, aerobics, balance games)	Walking program	2 months
	15/15						
Serino et al., 2017	20	20	3	4	NeuroVR (navigation within virtual city, discovering and memorizing the position of hidden objects)	Traditional cognitive rehabilitation (cognitive activities: cards games, naming, fluency, and music listening).	N/A
	10/10						

N/A, not available.

### Meta-analysis cognitive function

#### MoCA

**Figure 3:**
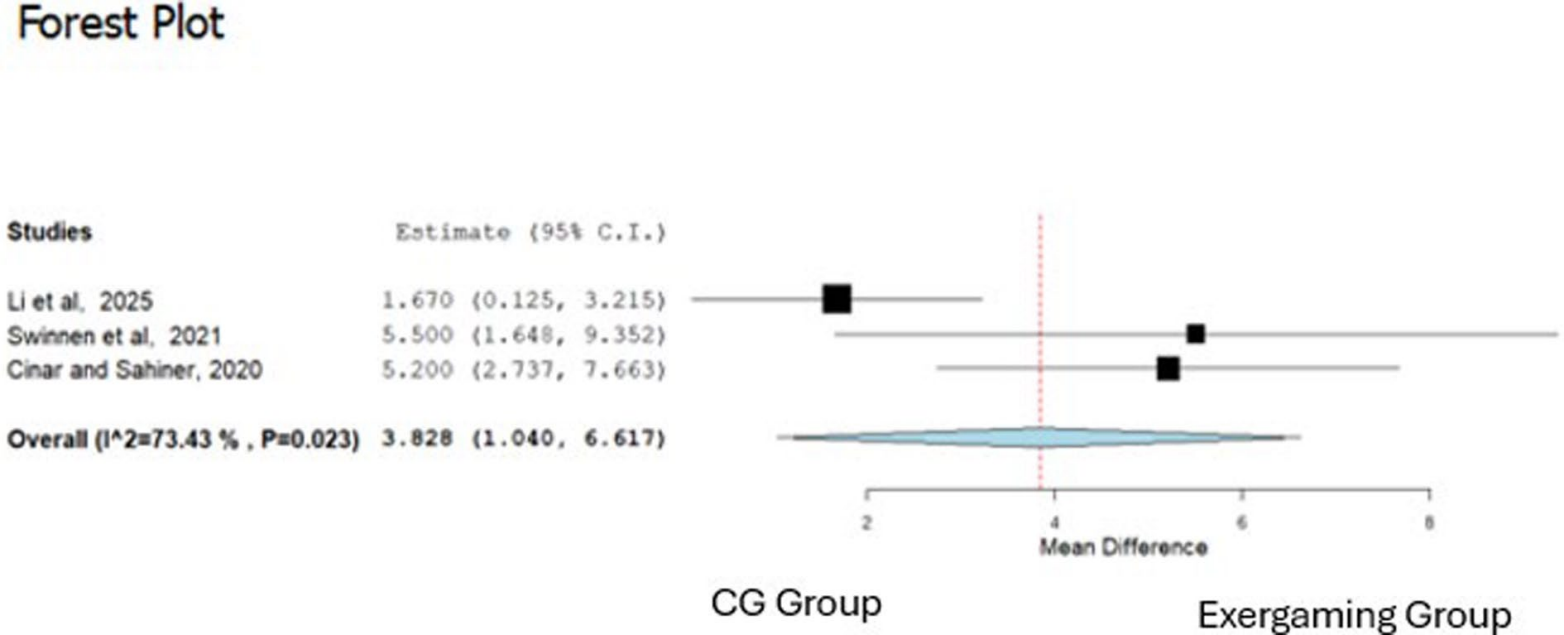
MoCA meta-analysis results.

Across the included trials, participants (*N* = 391) receiving gamified exergaming demonstrated significantly greater improvement in MoCA assessment compared to control groups. The mean difference (MD) was 3.83 (95% CI 1.04 to 6.61, *p* = 0.007), indicating a moderate and statistically significant advantage for the intervention. Between-study heterogeneity was substantial (*τ*^2^ = 4.32; *I*^2^ = 73.4%, *p* = 0.023), suggesting meaningful variability in effect sizes across studies. Inspection of the forest plot showed that two studies favored exergaming, while one study favored the control condition, although all confidence intervals crossed in the direction of the pooled effect. Overall, these findings suggest a positive but heterogeneous effect of exergaming on MoCA performance (Figure 3).

#### MMSE

**Figure 4:**
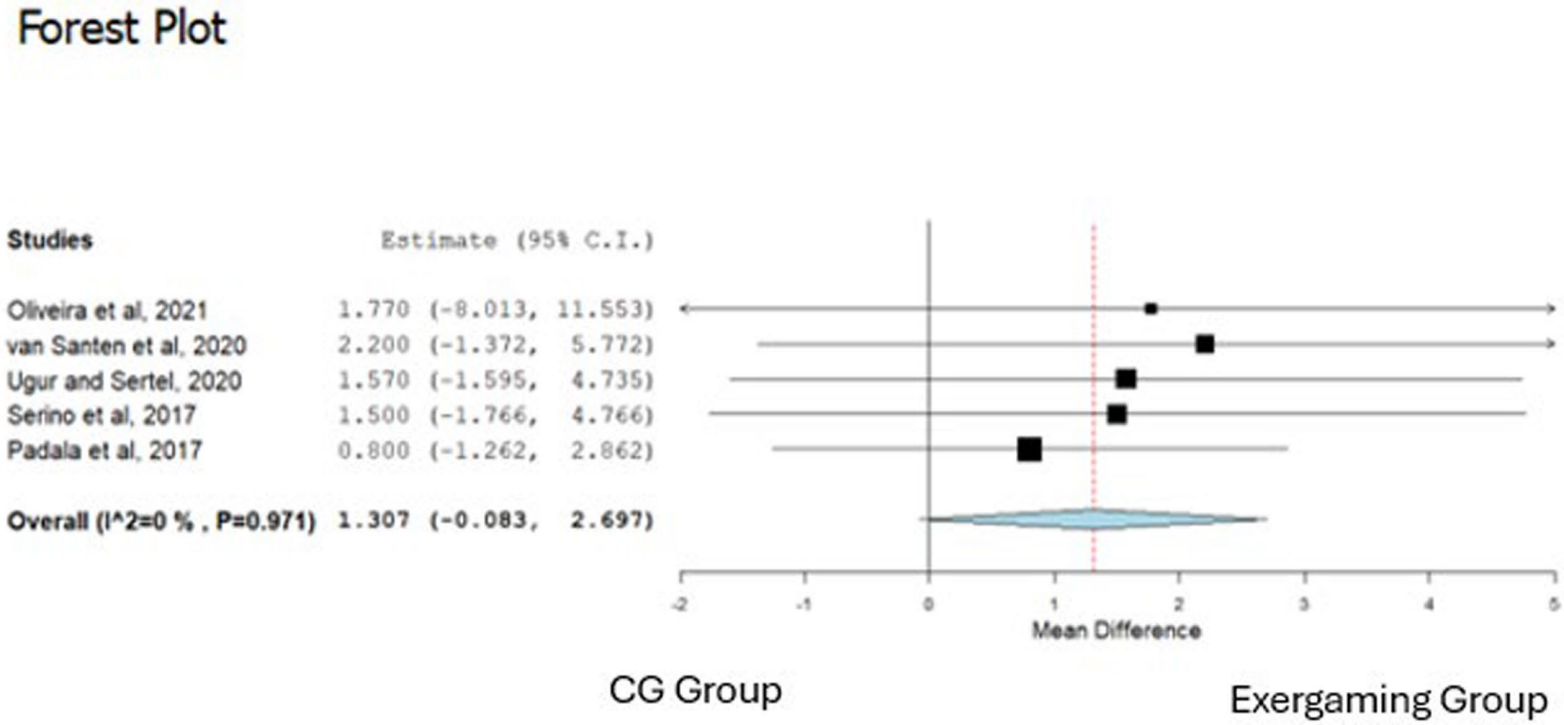
MMSE meta-analysis.

For the cognitive function measured with the Mini-Mental State Examination, the pooled analysis showed a non-significant difference between the two groups. The random (MD = 1.31) (95% CI –0.08 to 2.70, *p* = 0.065), indicating that gamified exergaming may confer small cognitive benefits, although the effect did not reach statistical significance. Heterogeneity across studies was absent (*τ*^2^ = 0.000; *I*^2^ = 0%, *p* = 0.971), suggesting highly consistent findings across trials (Figure 4).

### Meta-analysis ADL

#### Katz index

**Figure 5:**
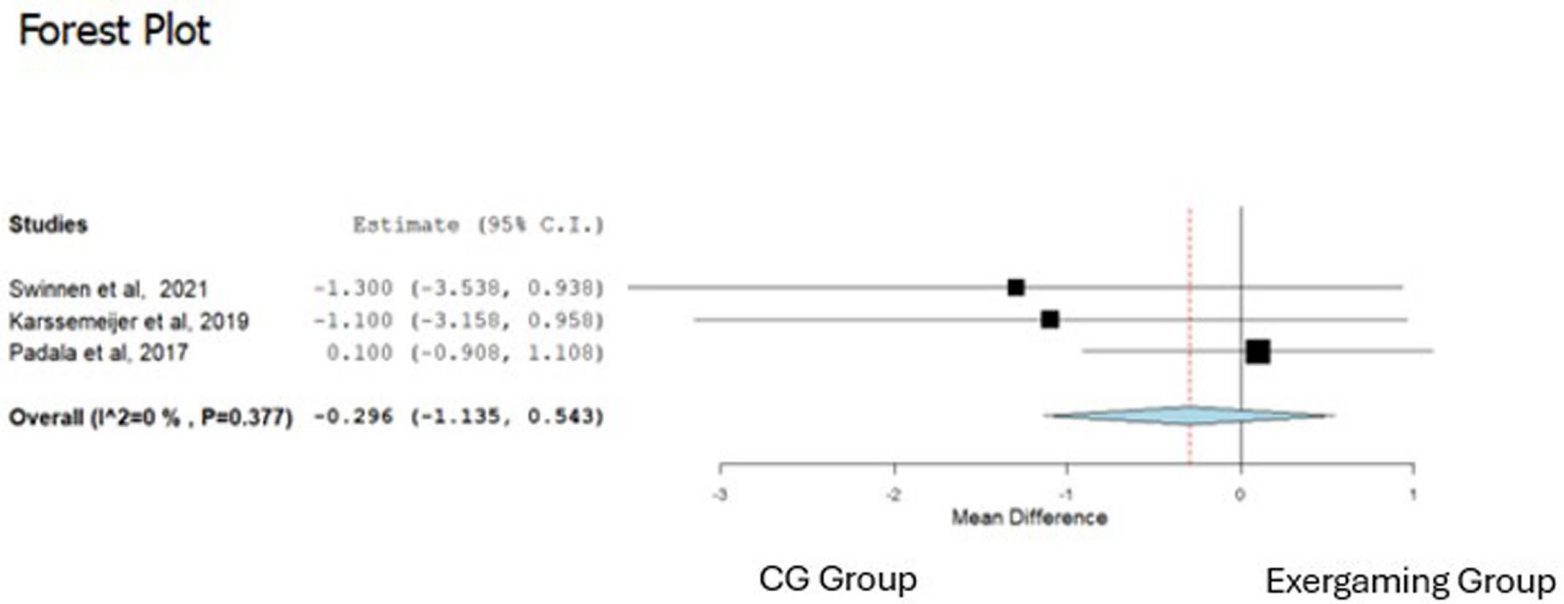
Katz ADL index.

The meta-analysis of the Activities of Daily Living with Katz index indicated no significant difference between the gamified exergaming intervention and control conditions. The model produced a MD = −0.29 (95% CI −1.14 to 0.54, *p* = 0.489), suggesting that the intervention did not meaningfully influence basic ADL performance. Heterogeneity across studies was absent (*τ*^2^ = 0.000; *I*^2^ = 0%, *p* = 0.377), indicating highly consistent findings (Figure 5).

### Meta-analysis motor function

#### SPPB

**Figure 6:**
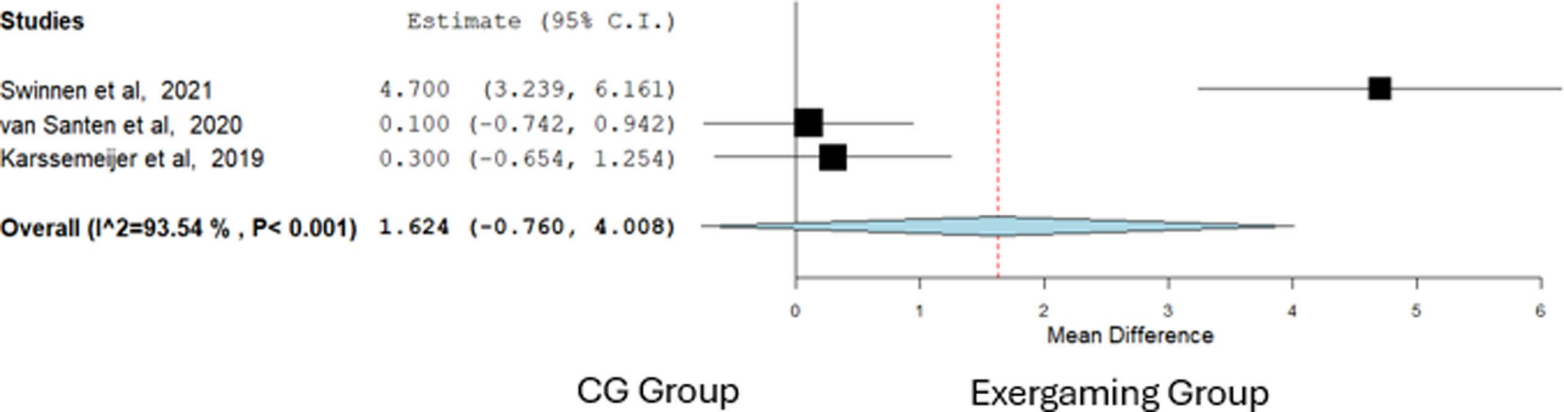
SPPB meta-analysis.

The meta-analysis of SPPB outcome yielded a pooled mean difference of 1.624 (95% CI: −0.760 to 4.008; *p* = 0.182), indicating that the overall effect did not reach statistical significance. However, the analysis revealed substantial between-study heterogeneity, with *I*^2^ = 93.54%, *Q* (2) = 30.97, and a heterogeneity *p*-value <0.001, suggesting considerable variability in effect sizes across the included studies. Performance, the aggregated evidence demonstrates inconsistent effects and high heterogeneity, limiting the certainty of the pooled estimate (Figure 6).

## Discussion

The findings of this systematic review and meta-analysis indicate that exergaming interventions provide measurable cognitive benefits for individuals with dementia compared with non-exergaming rehabilitation. The strongest effect was observed for global cognition assessed with the MoCA, which demonstrated significant improvement. This pattern aligns with evidence showing that cognitively enriched physical activity enhances executive functioning, attention, and processing speed more effectively than traditional exercise (Liao et al., 2019; Meekes and Stanmore, 2017). Dual-task physical–cognitive training may increase prefrontal and parietal network efficiency and promote neuroplasticity (Eggenberger et al., 2015), while the gamified structure of exergaming increases cognitive load through real-time decision-making, problem-solving, and coordinated movement. The interactive and multisensory nature of exergames may further stimulate neural networks and support cognitive engagement beyond conventional rehabilitation (Martin-Niedecken and Schättin, 2020).

In contrast, effects on MMSE did not reach statistical significance, although a positive trend was observed. This discrepancy is consistent with evidence that the MMSE is less sensitive to subtle cognitive changes in early to moderate dementia compared with the MoCA (Justo-Henriques et al., 2025). The divergence between these measures underscores the importance of selecting sensitive cognitive assessments when evaluating technology-based interventions (Jannati et al., 2024).

No significant improvements were observed in activities of daily living. This finding aligns with previous reviews reporting that, although exergaming may enhance cognitive engagement, its direct impact on functional independence is limited due to the multifactorial nature of functional decline in dementia (Swinnen et al., 2021; Werner et al., 2018). Many included studies did not target ADL as a primary outcome, and intervention duration or intensity may have been insufficient to produce functional change.

Motor outcomes also did not reach statistical significance, despite some individual studies reporting improvements in balance, gait speed, and mobility. Reduced motor learning capacity in dementia may limit the effectiveness of exergaming-based motor practice (Karssemeijer et al., 2019b; Voinescu et al., 2024). Although exergaming has shown motor benefits in older adults with neurodegenerative conditions (Skjæret et al., 2016; Teraz et al., 2022), the small number of dementia-specific trials and variability in motor outcome measures likely constrained the ability to detect consistent effects (Wu et al., 2023; Werner et al., 2018).

Interpretation of the present findings must remain grounded in evidence specific to dementia. Although research in MCI and other cognitively impaired populations provides useful context, these groups differ meaningfully from individuals with dementia in terms of neural plasticity, cognitive reserve, and tolerance for technologically mediated tasks (Osiurak and Reynaud, 2019). As a result, findings from immersive VR studies in other populations cannot be assumed to generalize to dementia. The present synthesis therefore focuses on exergaming-based interventions, predominantly non-immersive, screen-based systems with semi-immersive and fully immersive VR being minimally represented (Martin-Niedecken and Schättin, 2020; Voinescu et al., 2024). This imbalance limits the ability to determine whether higher immersion levels yield stronger cognitive or motor effects. Consequently, the pooled results should be interpreted as reflecting the general influence of exergaming rather than the specific contributions of individual VR modalities (Karaosmanoglu et al., 2025). References to mechanisms such as neuroplasticity, reward processing, or network efficiency are included only when supported by dementia-specific evidence or theoretical models relevant to dual-task physical and cognitive training (Bahar-Fuchs et al., 2019). Although more immersive systems may offer additional therapeutic potential, their feasibility, safety, and efficacy in dementia remain under-examined (Martin-Niedecken and Schättin, 2020). Future trials with adequate sample sizes and direct comparisons across immersion levels are required to clarify whether different technological formats produce differential therapeutic outcomes (Karaosmanoglu et al., 2025; Mehrabi et al., 2022). A clear distinction between exergaming and other VR modalities is therefore essential to avoid over-generalization and ensure that conclusions reflect the scope and limitations of the available dementia research (Zhao et al., 2020; Chan et al., 2024).

Overall, the evidence suggests that exergaming may serve as a feasible and engaging adjunct to support cognitive function in dementia, particularly when interventions incorporate meaningful cognitive challenges. However, the current evidence base does not support strong conclusions regarding functional or motor benefits. The magnitude of cognitive improvement in dementia also appears smaller than that reported in MCI, consistent with meta-analytic findings indicating stronger effects in individuals with milder impairment (Kim et al., 2019; Chan et al., 2024). Differences in neural plasticity, task engagement, and usability demands may contribute to this pattern. As dementia prevalence continues to rise, exergaming represents a promising, engaging, and scalable therapeutic option that may complement traditional rehabilitation approaches (Karssemeijer et al., 2019b; Voinescu et al., 2024).

### Limitations

Confidence in the present findings is constrained by several methodological limitations. The number of randomized controlled trials conducted specifically in dementia populations remains small, reducing statistical power and contributing to the non-significant results observed for MMSE, ADL, and motor outcomes (Zhu et al., 2022; Devanand et al., 2022). Considerable heterogeneity in intervention protocols including differences in duration, frequency, intensity, and technological platforms, further limits comparability across studies (Zhao et al., 2020; Zhu et al., 2022; Soleimani et al., 2024; Cassani et al., 2020). Variability in outcome measures, particularly in the motor and functional domains, also restricts the ability to detect consistent effects, as studies employed diverse assessment tools with limited standardization (Maggio et al., 2025b; Perry et al., 2019; Negm et al., 2019; Pavasini et al., 2016).

Generalizability is additionally limited by the predominance of non-immersive exergaming systems, whereas more immersive VR modalities that have been shown to produce stronger cognitive effects in other populations remain under-represented in dementia research (Liepa et al., 2022). Variation in dosage reporting, including inconsistent documentation of active gameplay time versus total session duration, further complicates interpretation of dose–response relationships. Finally, the absence of long-term follow-up data prevents conclusions about the durability of cognitive or motor improvements.

### Recommendations for future studies

Future research should prioritize larger, well-designed randomized controlled trials that directly compare exergaming with conventional rehabilitation in dementia populations. Standardizing intervention parameters such as dosage, frequency, and progression would improve comparability and support the development of evidence-based guidelines (Karssemeijer et al., 2019b; Wu et al., 2025). Researchers should also adopt uniform cognitive, motor, and functional outcome measures, enabling more robust meta-analytic synthesis (Burridge et al., 2019). Given the promising cognitive effects observed in this review, future studies should explore the mechanisms underlying cognitive-motor engagement in exergaming and examine whether immersive VR platforms can safely enhance therapeutic outcomes in dementia (Van Santen et al., 2018). Finally, studies with extended follow-up periods are essential to determine whether gains in cognition or mobility translate into sustained improvements in daily functioning and quality of life (Bamidis et al., 2015; Marusic et al., 2018).

## Conclusion

This systematic review and meta-analysis indicate that exergaming may offer cognitive benefits for individuals with dementia, with the most consistent improvements observed in global cognition when assessed using more sensitive measures such as the MoCA. In contrast, effects on MMSE, motor performance, and activities of daily living were less consistent or non-significant, and therefore no firm conclusions can be drawn regarding these domains. When compared with non-exergaming interventions, exergaming did not demonstrate clearly superior effects across all outcomes, although it may provide added cognitive stimulation through its interactive and engaging format.

Given the limited number of trials, the heterogeneity of intervention protocols, and inconsistent reporting of safety, adherence, and acceptability, the clinical relevance and real-world applicability of exergaming remain uncertain. Exergaming should therefore be viewed as a potential adjunct, rather than an established therapeutic approach, in dementia rehabilitation. Further trials with clear reporting of exposure time, adverse events, and long-term follow-up are needed to determine whether exergaming can reliably support cognitive functioning and functional well-being in people living with dementia.
